# Systematic Analysis of Cold Stress Response and Diurnal Rhythm Using Transcriptome Data in Rice Reveals the Molecular Networks Related to Various Biological Processes

**DOI:** 10.3390/ijms21186872

**Published:** 2020-09-19

**Authors:** Woo-Jong Hong, Xu Jiang, Hye Ryun Ahn, Juyoung Choi, Seong-Ryong Kim, Ki-Hong Jung

**Affiliations:** 1 Graduate School of Biotechnology & Crop Biotech Institute, Kyung Hee University, Yongin 17104, Korea; hwj0602@khu.ac.kr (W.-J.H.); kangwuk97@khu.ac.kr (X.J.); ahyeryun@naver.com (H.R.A.); 2 Department of Life Science, Sogang University, Seoul 04107, Korea; cjy1835@naver.com

**Keywords:** rice, cold stress response, circadian clock, diurnal rhythm, transcriptome analysis, *sgr* mutant

## Abstract

Rice (*Oryza sativa* L.), a staple crop plant that is a major source of calories for approximately 50% of the human population, exhibits various physiological responses against temperature stress. These responses are known mechanisms of flexible adaptation through crosstalk with the intrinsic circadian clock. However, the molecular regulatory network underlining this crosstalk remains poorly understood. Therefore, we performed systematic transcriptome data analyses to identify the genes involved in both cold stress responses and diurnal rhythmic patterns. Here, we first identified cold-regulated genes and then identified diurnal rhythmic genes from those (119 cold-upregulated and 346 cold-downregulated genes). We defined cold-responsive diurnal rhythmic genes as CD genes. We further analyzed the functional features of these CD genes through Gene Ontology and Kyoto Encyclopedia of Genes and Genomes enrichment analyses and performed a literature search to identify functionally characterized CD genes. Subsequently, we found that light-harvesting complex proteins involved in photosynthesis strongly associate with the crosstalk. Furthermore, we constructed a protein–protein interaction network encompassing four hub genes and analyzed the roles of the *Stay-Green (SGR)* gene in regulating crosstalk with *sgr* mutants. We predict that these findings will provide new insights in understanding the environmental stress response of crop plants against climate change.

## 1. Introduction

Rice (*Oryza sativa* L.) is a model crop plant and a staple crop across the globe, particularly Asia. Owing to dramatic global climate change and population growth, it has become necessary to increase the production of staple food sources [1,2]. Because rice is a cold-sensitive crop, cold stress encompassing chilling (0–20 °C) and freezing temperature (below 0 °C) is considered a major factor that leads to drastic reduction in rice productivity [3,4,5]. Therefore, various studies on enhancing cold stress tolerance have been reported, with the identification of important factors affecting cold stress response in rice [6]. However, there is evidence that the cold stress response is associated with various biological processes, including biological clocks, disease resistance, metabolism, hormonal balance, and aging [7,8,9]. This complexity is not only a major obstacle to increasing cold stress tolerance but also to the regional adaptation of resistant varieties.

The circadian clock caused the diurnal rhythmic expression of the genes to cope with the earth’s rotation and daily repeated environmental fluctuations. Organisms have evolved adaptive mechanisms associated with the light/dark environmental alternations and temperature changes, which manifests as a profound change in metabolism, physiology, and behavior occurring between the day and night cycles in most organisms [10,11]. Circadian clock greatly affect plants, particularly regarding synchronizing biological processes and increasing the efficiency of photosynthesis owing to the non-motile feature of these organisms [12,13,14]. It is involved in various physiological regulatory roles, including flowering time, by controlling the expression and photostability of CONSTANS proteins, which induce FLOWERING LOCUS proteins for flowering [15].

With the recent introduction of high-throughput technology such as microarrays, several reports provided insights into the molecular components of the crosstalk between the circadian clock and cold stress response [16]. There are reports on Arabidopsis that the disruption of the circadian clock changes cold-response-associated transcriptomes, and the knockout of the three pseudo-response regulator genes (PRR), *PRR5*, *PRR7*, and *PRR9,* affects the expression of stress response genes [17,18]. Several cold-related and circadian rhythm genes have been reported in rice. A serine-threonine protein kinase without lysine kinase, *OsWNK1*, exhibited differential expression under various abiotic stressors, including cold, and also exhibited circadian rhythm [19]. Aquaporin in rice roots is responsible for cold stress-induced acclimation and a cold-induced MYB transcription factor, *CMYB1*, exhibited a circadian rhythm expression pattern in rice leaves [20,21]. However, the crosstalk between cold stress response and the circadian clock in rice remains unclear from a systems biology approach.

To understand the interplay between the circadian clock and cold stress response in rice, we performed a systemic analysis using two large transcriptome datasets related to the diurnal rhythm and abiotic stress such as drought, salt, cold, and submergence. We identified cold-regulated genes and then identified cold-responsive diurnal rhythmic genes (hereafter CD genes; 119 and 346 genes for up/down-regulation, respectively). Next, we analyzed the functional features of these CD genes through Gene Ontology (GO) and Kyoto Encyclopedia of Genes and Genomes (KEGG) enrichment analysis, after a literature search for functionally characterized genes. Furthermore, we constructed a protein–protein interaction (PPI) network and validated it using the *stay-green* (*sgr*) mutant for the regulation of the crosstalk between the cold-response and circadian clock. Based on these results, we proposed that a hypothetical molecular network mediates interplay among various biological processes, including the circadian clock, cold stress response, hormone signaling, and senescence. Our systems biology approach regarding the cold stress response and circadian clock identified some details of the molecular network which could be used to improve the productivity and regional adaptation of rice.

## 2. Results

### 2.1. Genome-wide Identification of Cold Stress Response Genes Exhibiting Diurnal Rhythm Expression Patterns Using Meta-Expression Datasets

**Figure 1:**
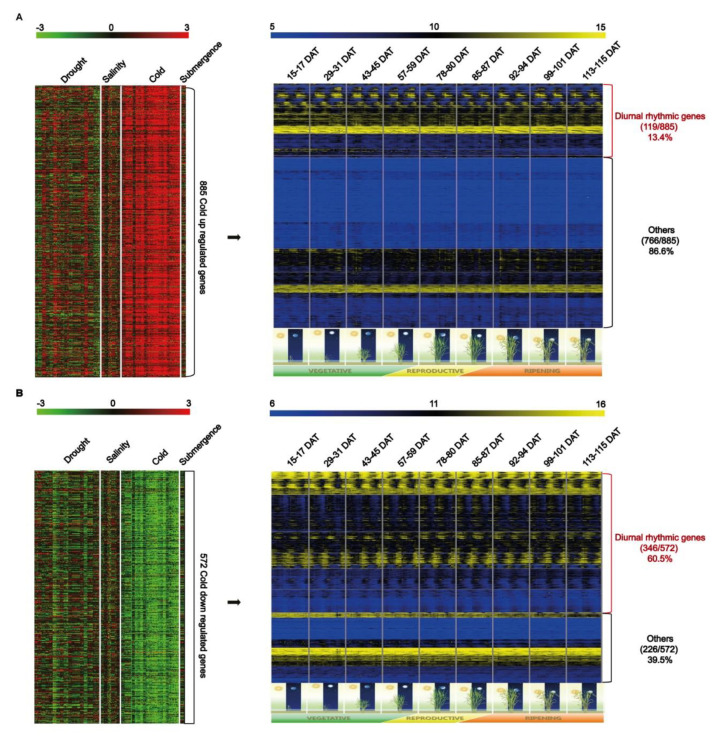
Heat map of cold-regulated genes with diurnal rhythmic expressions. (**A**) Overview of the process that identified 885 cold-upregulated genes, 119 of which were shown to have diurnal rhythmic expression. (**B**) Overview of the process that identified 572 cold-downregulated genes, 346 of which were shown to have diurnal rhythm expression. Cold-responsive diurnal rhythm genes are indicated in red. DAT means days after transplanting [22].

For systematic analyses aimed at revealing the unknown molecular mechanisms of cold stress response and circadian rhythm, we identified candidate genes responsive to cold stress and diurnal rhythm expression patterns (hereafter cold-responsive diurnal rhythmic genes; CD genes). We identified rice genes unique to cold stress response using a meta-expression dataset consisting of four abiotic stress responses such as drought, salt, cold, and submergence. Consequently, we identified 885 and 572 cold-response genes that were up/downregulated at least 2-fold, respectively (Table S1). Next, we then analyzed the diurnal expression patterns of these genes using Agilent 44K array data of rice leaves under natural field conditions, encompassing the vegetative, reproductive, and ripening stages, at 2-h intervals for 2 days in each developmental stage [22]. Finally, we identified 119 and 346 up/downregulated CD genes, respectively, exhibiting diurnal rhythm expression patterns (Figure 1, Table S2). These genes were used for further analyses.

### 2.2. Literature Analysis to Identify Characterized Gene Functions Associated with CD Genes

To explore the CD genes’ functional roles, we retrieved information of functionally characterized genes from the Overview of functionally characterized Genes in Rice Online database (OGRO, http://qtaro.abr.affrc.go.jp/ogro/table) [23]. Regarding upregulated CD genes, the functions of 14 of these have been reported. Four genes (*basic leucine zipper 52* (*OsBzip52*), *rice carbon catabolite repressor 4-associated factor 1B*, *mitogen-activated protein kinase 5 (OsMAPK5)*, and *dehydration-responsive element-binding protein 1A*)) are related to cold stress tolerance [24,25,26,27]. One (*calcium-dependent protein kinase 18*) is related to blast resistance [28]. One (*Stress-responsive NAC1*) is related to drought tolerance [29]. Three (*OsBZR1, OsDWARF*, and *TIFY11b*) are related to dwarfism [30,31,32]. Two (*Ethylene response 2* and *rice Dof daily fluctuations 1* (*OsRdd1*)) are related to flowering time [33,34]. Two (*Jumonji C domain-containing protein 6* and *Multi-floret spikelet1*) are related to floral organ identity [35,36]. Lastly, one (*SGR*) is related to leaf senescence [37]. Furthermore, we identified 13 downregulated CD genes with known functions from the OGRO database. Two genes (*histone deacetylase 701* and *OsTFX1*) are related to bacterial blight resistance [38,39]. Two (*OsLSD1* and *stromal-derived factor-2-1*) are related to blast resistance [40,41]. One (*calcineurin B-like protein-interacting protein kinase 12*) is related to drought tolerance [42]. Three (*Cytokinin-responsive GATA transcription factor 1, histone deacetylase 702,* and *ascorbate peroxidase 2*) are related to dwarfism [43,44,45]. One (*waxy*) is related to seed amylose content [46]. One (*OsCDT3*) is related to aluminum tolerance [47]. One (*days to heading on chromosome 8*) is related to flowering time [48]. Two (*faded green leaf* and *zebra-necrosis*) are related to chlorophyll biosynthesis [49,50]. This result means that cold-upregulated genes are more significant targets for cold stress tolerance than downregulated genes. Moreover, this indicated that the CD genes are not only related to abiotic stress, including low temperature, but also to various other processes such as biotic resistance, senescence, and development (Table 1).

### 2.3. GO and KEGG Enrichment Analyses of the CD Genes Reveal that Photosynthesis and Light Harvesting Are Closely Related to the Cold Stress Response and Circadian Clock

**Figure 2:**
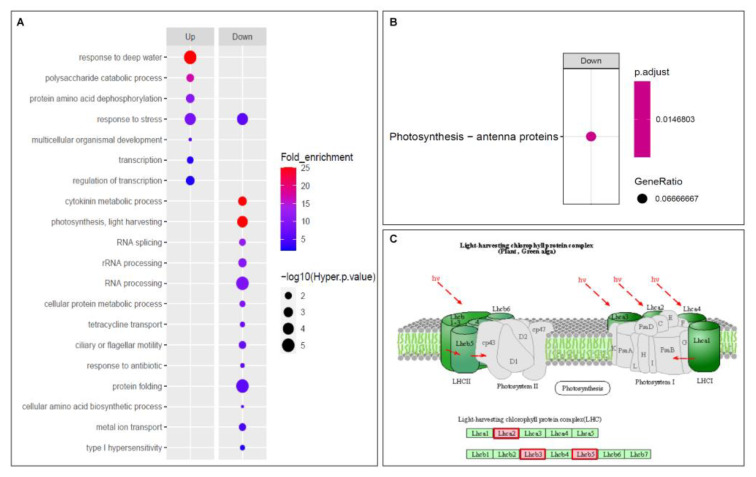
Functional classification of the CD genes via GO and KEGG analysis. (**A**) GO enrichment within the biological process category; terms were identified for 465 candidate genes. Dot colors indicate fold-enrichment values (blue color indicates 2-fold, which is the minimum cut off to select significant fold-enrichment value, and red color indicates fold-enrichment values greater than 2), and dot size indicates statistical significance (−log10(hyper *p*-values) are used, with higher values having greater significance). (**B**) KEGG enrichment analysis of candidate genes. Enriched KEGG pathways are indicated, with dot size representing the ratio of selected genes to total genes in the pathway, and dot color illustrating adjusted *p*-values. (**C**) Overview of the light-harvesting chlorophyll protein complex associated with downregulated CD genes visualized with the KEGG mapper webtool (http://www.genome.jp/kegg/mapper.html). Red colored boxes indicate mapped subunits of light-harvesting chlorophyll protein complex.

To further explore the biological significance of genes involved in the crosstalk between circadian clock and the cold stress response, we conducted GO and KEGG enrichment analyses. In total, 21 GO terms from the biological process were enriched for up/downregulated CD genes with the criteria of hypergeometric *p*-value of < 0.05 and fold-enrichment value of > 2 (Figure 2A, Table S3). The seven GO terms of upregulated CD genes were as follows: response to deep water (192.3-fold enrichment value, GO:0030912); polysaccharide catabolic process (17.0, GO:0000272); protein amino acid dephosphorylation (10.6, GO:0006470); response to stress (8.5, GO:0006950); multicellular organismal development (5.7, GO:0007275); transcription (2.7, GO:0006350); and regulation of transcription (2.5, GO: 0045449). GO enrichment analysis of the downregulated CD genes revealed that 14 biological processes were overrepresented. These processes included: the cytokinin metabolic process (27.5-fold enrichment value, GO:0009690); photosynthesis and light harvesting (26.8, GO:0009765); RNA splicing (11.7, GO:0008380); rRNA processing (10.3, GO:0006364); RNA processing (9.2, GO:0006396); cellular protein metabolic process (8.9, GO:0044267); tetracycline transport (7.4, GO:0015904); ciliary or flagellar motility (7.2, GO:0001539); response to antibiotics (6.3, GO:0046677); protein folding (6.0, GO:0006457); response to stress (5.9, GO:0006950); cellular amino acid biosynthetic process (5.6, GO:0008652); metal ion transport (4.7, GO:0030001); and type 1 hypersensitivity (2.7, GO:0016068).

In addition to GO enrichment, we performed KEGG enrichment analysis to identify the metabolic pathways involved in cold response and the circadian clock mechanism. Interestingly, only one pathway, photosynthesis-antenna proteins, was enriched, which was consistent with the enriched GO term in downregulated CD genes (Figure 2B). Using the KEGG mapper software, we identified three light-harvesting proteins involved in this pathway (Figure 2C) [51]. These results were consistent regarding photosynthesis and the light harvesting process mainly associated with the cold stress response and circadian clock, which was also confirmed with the MapMan software (3.6.0 RC1; Figure S1A, Table S4). Along with the photosynthesis process, GO and MapMan results also implied a variety of potential biological processes involved in this crosstalk (Figure S1B).

### 2.4. Construction of Protein-protein Interactions of CD Genes Reveals the Molecular Network Interplay with Various Biological Processes

**Figure 3:**
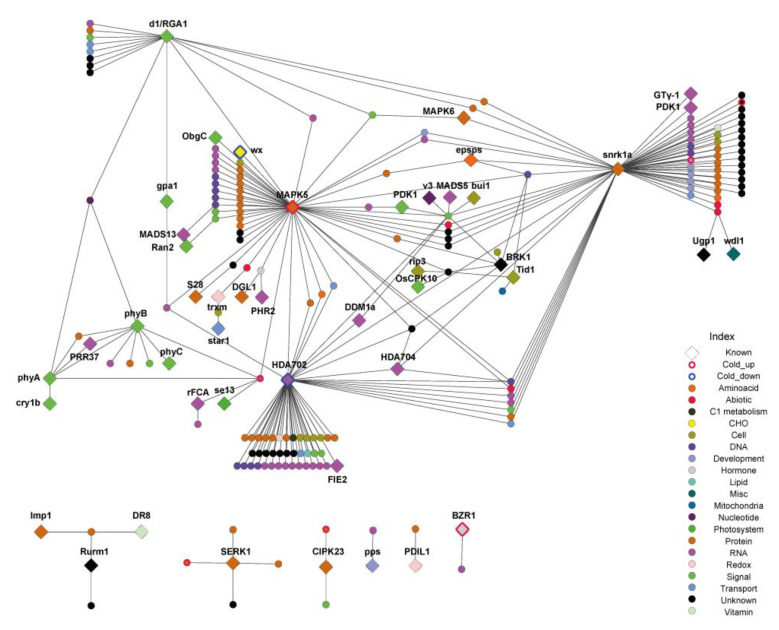
Hypothetical protein–protein interaction (PPI) network associated with CD genes. The PPI network constructed from the Rice Interactions Viewer by querying all CD genes. The network was simplified by removing all genes that were not functionally characterized. The full network is shown in Figure S2.

To further explore the molecular mechanisms regulating crosstalk between the circadian clock and cold stress response, we constructed a hypothetical protein–protein interaction (PPI) network of up/downregulated CD genes using a rice interaction viewer (http://bar.utoronto.ca/interactions/cgi-bin/rice_interactions_viewer.cgi) [52]. This viewer generated a network of CD genes comprising 764 nodes and 1340 edges (Figure S2). However, owing to its complexity, we reconstructed the network to only show the interactions between genes for which some information was available. The reconstructed network comprised 208 nodes and 248 edges (Figure 3, Table S5), including both up- and downregulated CD genes.

Among the upregulated CD genes, seven were present in the network nodes, two of which were functionally characterized. *Brassinozole-resistant1* (*OsBZR1*) is a key transcription regulator in rice with activity regulated by 14-3-3 interactions. The other gene is a *mitogen-activated protein kinase in rice* (*OsMAPK5*) that was reported as an ABA-induced regulator that can modulate disease resistance and abiotic stress tolerance [26,30]. Among the downregulated CD genes, two known genes were identified in the network nodes: the *waxy* gene in rice (*Wx*), which is associated with the amylose content in rice endosperms, and the *rice histone deacetylase* gene (*OsHDA702*), which is associated with plant growth, particularly in the root [44,46]. Among the interactors, 42 known genes were identified, five of which were related to flowering time, including *phytochromes A, B, and C* (*OsPhyA, OsPhyB, OsPhyC*); four were related to biotic stress resistance, including *calcium-dependent protein kinase 10* (*CPK10*), *somatic embryogenesis receptor-like kinase 1* (*SERK1*), *WRKY62*, and *disease resistance-responsive gene 8* (*DR8*); four were related to dwarfism, including *decreased DNA methylation* (*DDM1a*), *dwarf 1* (*D1*), *pyruvate dehydrogenase kinase 1* (*PDK1*), and *HDA704*; and three were related to chloroplast development, including *virescent3* (*V3*), *thioredoxin m* (*Trxm*), and *spo0B-associated GTP-binding protein* (*ObgC*). *Flowering time control gene in rice* (*rFCA*) and *pseudo-response regulator* (*OsPRR37*) in rice were also identified. Other identified genes are listed in Table 2.

### 2.5. Validation of the Cold Stress Response and Circadian Clock Network with a Case Study Using the Stay-green (sgr) Mutant

**Figure 4:**
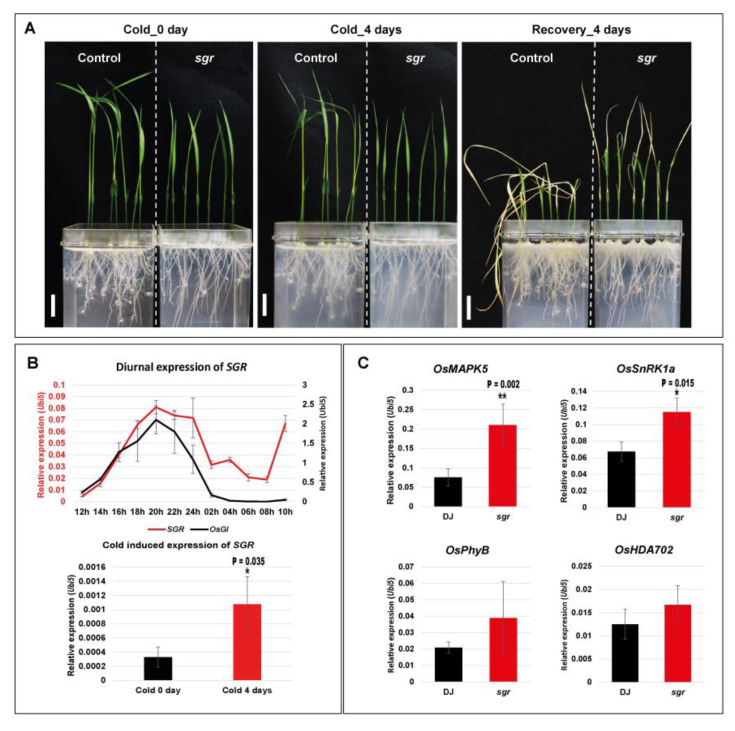
Phenotypes of the *sgr* mutant compared with wild-type controls under cold stress, and the role of *SGR* in the regulatory network. (**A**) Phenotypic comparison of DJ and *sgr* during cold stress. Bar = 2 cm. (**B**) Validation of the differential expression patterns of the *SGR* gene under cold stress and diurnal rhythm. *OsGI*, *rice gigantea* gene used as a diurnal rhythm marker gene showing a peak in the evening. Y-axis colors indicate relative expression value of *SGR* (red) and *OsGI* (black) to *OsUbi5,* respectively. (**C**) Expression levels of the cold-stress hub genes *OsMAPK5*, *OsSnRK1a*, *OsPhyB*, and *OsHDA702* in the leaves of DJ and *sgr* mutants after 4 days of cold treatment. DJ, Dongjin; *sgr, SGR* defective mutant. * *p* < 0.05; ** *p* < 0.01.

Among the functionally characterized genes exhibiting crosstalk between cold stress response and circadian clock, *sgr* mutant maintains greenness during leaf senescence [37]. Under cold stress for 4 days and recovery for 4 days, we found that the *sgr* mutant exhibited more cold stress tolerant phenotype than the control plant, Dongjin rice (DJ) (Figure 4A). Moreover, we confirmed that this tolerant phenotype is not originated from the developmental effect using reproducible experiment with Hwacheong-wx rice in the same genetic background with *sgr* mutant (Figure S3). Upregulation of the *SGR* gene under cold treatment and its diurnal expression patterns were confirmed by qRT-PCR (Figure 4B). The expression patterns of the four hub genes in our PPI network, i.e., *OsMAPK5* (LOC_Os03g17700), *OsSnRK1a* (LOC_Os05g45420), *OsPhyB* (LOC_Os03g19590), and *OsHDA702* (LOC_Os06g38470) (Figure 4C) were then examined. All four genes were upregulated in the *sgr* mutants compared with DJ after cold treatment for 4 days. Two genes were also significantly changed. Consistent with the results of our PPI network analysis, *sgr* mutants also exhibited altered expression levels of four hub genes in response to cold stress. These results suggest that several biological processes, including cold response and senescence, are linked through the hub genes predicted by our PPI network model.

## 3. Discussion

Cold threatens the normal growth and yield requirements of various major crops [92]. The circadian clock rhythmically controls the behavior and physiological activities of plants for improved environmental adaptation [16,93]. The interplays between the circadian clock, light-quality, and cold-response have been studied intensively in the model plant, Arabidopsis thaliana [13,94]. Circadian clock components such as *CIRCADIAN CLOCK ASSOCIATED 1*, *TIMING OF CAB EXPRESSION1*, *LATE ELONGATED HYPOCOTYL* and *Pseudo-Response Regulators* (*PRR*) genes mutually interact with temperature signals [95]. Moreover, the photoreceptors: ZEITLUPE, phytochromes, and cryptochromes which are regulated by GIGANTEA protein, integrate light-quality information with circadian clock [96,97,98,99]. These clock components have a role in regulating the expression of C-repeat binding factors that bind to the promoter of the cold-regulated genes [100]. In rice, the circadian clock genes have been reported using ortholog search, but it has been mainly studied in depth related to the agronomic traits especially, flowering time [101]. The research of the trait along with the geographical distribution of the rice suggested the relationship between the circadian clock and cold stress [67], but, still, there have been limited reports to shape the molecular network as in the arabidopsis. 

Therefore, it is of great developmental significance to explore the possible correlations between cold stress response and circadian clock through systematic analysis in rice.

We first identified 465 CD genes, including 119 upregulated and 346 downregulated genes. These were identified through global transcriptome analyses using public resources, which provided a more extensive scope of utilities that could possibly enhance cold tolerance in rice. These genes will serve as the basis for a series of studies to follow.

To understand the meaningful biological processes underlying the crosstalk between cold stress response and circadian clock in rice, we performed GO and KEGG enrichment analyses. Based on the visualized results, we found that photosynthesis and light harvesting are key biological processes in rice associated with cold stress response. Previous studies also suggested the importance of photosynthesis in cold stress response and circadian clock. For instance, several genes of Zea mays cold-induced genes associated with photosynthesis during long-term cold treatment in maize seedlings [102]. Transgenic rice plants overexpressing *choline oxidase A* exhibited expanded Fv/Fm when exposed to cold stress [103]. Besides, some researchers indicated that photosynthesis is coupled with diurnal rhythms through light harvesting and CO2 fixation rates by ribulose 1,5-bisphosphate carboxylase/oxygenase (Rubisco) [104]. However, research on photosynthesis and the crosstalk effects of cold stress and circadian clock remains limited. We also found that the GO term ‘polysaccharide catabolic process’ was highly enriched in the upregulated CD genes. This could be explained by the energy supply required for plants under cold stress.

Our network analysis suggests a hypothetical molecular mechanism underlying the diverse phenomenon related to crosstalk between cold stress response and circadian clock regulation. In this network, *OsPhyA*, *OsPhyB*, *OsPhyC*, and *OsPRR37* are key components associated with the regulation of circadian clock. These components interact with both *OsMAPK5*, a component of Ca^2+^ signaling and modulator of disease resistance that is upregulated in response to cold and exhibits diurnal rhythmic expression, and *OsHDA702*, a component of DNA remodeling that is downregulated in response to cold and exhibits rhythmic expression [44,105]. *OsSnRK1a* has roles in regulating various metabolism-related processes in plants and is also related to ABA signaling [106,107]. Moreover, *D1/RGA1* is related to these four hub genes. Studies of *daikoku 1 (d1)* mutant using microarray analysis revealed that *D1*/*RGA1* has a role in regulating multiple abiotic stresses, including drought, cold, heat, and salinity [108]. Angel et al. (2016) also revealed decreased drought sensitivity in the erect leaves of *d1* mutant owing to the increased photo-avoidance and more effective light harvesting [109,110]. More interestingly, *sgr* mutant with cold tolerant phenotype is exhibited as erect leaves which is similar to *d1* mutant, and the expression level of four hub genes are all upregulated in *sgr* mutant relative to wild type. Thus, the identification of these components implies that cold-induced *SGR* and four hub genes (*OsMAPK5*, *OsSnRK1a*, *OsHDA702*, and *OsPhyB*) might coregulate the physiological response to cold in a circadian clock-dependent manner.

**Figure 5:**
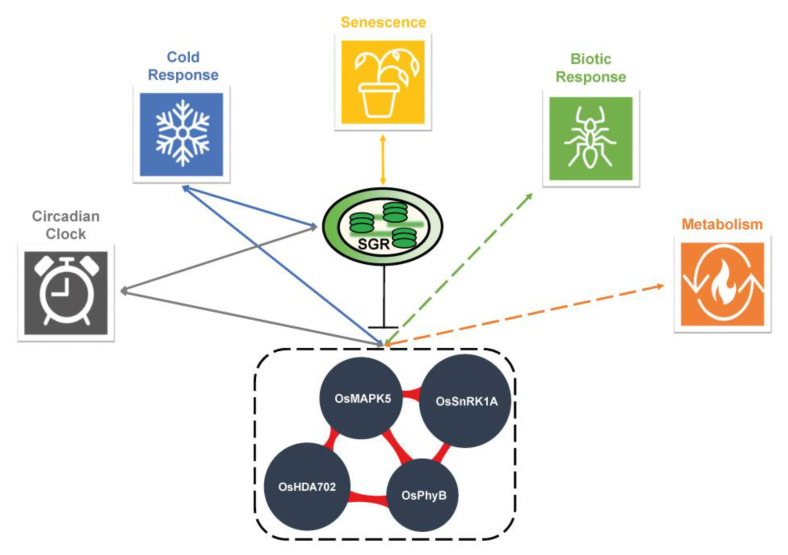
Hypothetical model of the crosstalk among different biological processes mediated by the cold stress hub genes. Different biological processes are thought to be linked via OsMAPK5, OsSnRK1a, OsPhyB, and OsHDA702 as intermediates. The arrow means confirmed interplay with our experimental validation and dashed one means postulated interplay from the literature study.

Overall, we hypothesize that the interconnection between different physiological responses, such as cold, and circadian clock is achieved by manipulating the expression of the hub genes (*OsMAPK5*, *OsSnRK1a*, *OsHDA702*, and *OsPhyB*). Cumulatively, we constructed a framework diagram of multiple elements that are interconnected through the hub genes (Figure 5). More experimental evidence is required to validate this hypothesis; however, we anticipate that our data will serve as a bridge for integrating initial studies on cold stress and circadian clock.

## 4. Materials and Methods

### 4.1. Collection of Microarray Data and Meta-expression Analysis

To understand the crosstalk between cold stress and circadian clock regulation, we used two different meta-expression datasets: Affymetrix array gene expression dataset for abiotic stresses (E-MEXP-2401, GSE16108, GSE18930, GSE21651, GSE24048, GSE25176, GSE26280, GSE33204, GSE37940, GSE38023, and GSE6901) and a diurnal dataset comprising Agilent 44K array gene expression data from 202 leaf samples collected from nine developmental stages (GSE36040). As previously described [111,112], public transcriptome data were downloaded from the GEO database [113]. After collecting the expression datasets, data were normalized with the Affy and Limma packages using the R programming language [114,115]. After normalization, intensity values were converted to log2 scale, and the log2 fold-change values in response to cold stress (compared with their values in an untreated control) were calculated. Cold responsive genes were defined as those with a ≥ 1.5 or ≤−1.5 log2 fold change, and a *p*-value of ≤ 0.05. For the selected genes, log2 fold-change data were visualized with those in other abiotic stress treatments using the MeV software [116]. KMC clustering in MeV was used to identify cold-induced or cold-repressed diurnal rhythmic genes.

### 4.2. Literature Search for Functionally Characterized Genes

The OGRO database was searched to determine the functionally characterized cold-induced diurnal rhythmic genes [23]. Detailed information is presented in Table 1.

### 4.3. GO and KEGG Enrichment Analyses

The rice oligonucleotide array database was used to retrieve GO information for our candidate genes [117]. Fold-enrichment values were obtained by dividing the query number by the query expected value. Significant GO terms were defined as those with a fold-enrichment value of >2 and a hypergeometric *p*-value of <0.05. The ClusterProfiler software package was then used to perform KEGG enrichment analysis [118]. To use the enrichKEGG function in this package, we used input data containing clustering information and the ID of the rice annotation project database. Additionally, we selected an adjusted cutoff *p*-value of <0.05, thereby selecting the organism code data and filtering the results. To visualize these results, we used the dotplot function of R studio (version: 1.2.5042) and modified the graph using the ggplot2 software package (version: 3.3.0). Illustrator software was used to refine the presentation.

### 4.4. MapMan Analysis

We used the MapMan toolkit (3.6.0 RC1) to functionally classify our candidate genes [119]. We assigned different colors for diurnal rhythmic genes that were up/downregulated (up, red; down, green) in response to cold stress and visualized all candidate genes simultaneously. In our analysis, we used metabolism and regulation overviews. Detailed information regarding the MapMan analysis is presented in Table S4.

### 4.5. Protein–Protein Interaction Network Construction

A hypothetical protein–protein interaction network was generated to investigate crosstalk between the cold stress response and circadian rhythm. A network file of the queried genes (up/down-CD genes) was obtained from the rice interaction viewer (http://bar.utoronto.ca/interactions/cgi-bin/rice_interactions_viewer.cgi). Next, a base file containing interaction information was constructed. A data file containing additional information on network nodes was also generated. These data were visualized using the Cytoscape software (3.3.0) [120,121]. We used a *p*-value threshold of <0.05 and enabled the “fusion option”, which merges nodes with similar information to simplify our network.

### 4.6. Plant Material and Stress Treatment

Japonica rice (*Oryza sativa* L.) (cv. Dongjin and Hwacheong-wx) seeds and *sgr* defective mutants (cv. Hwacheong-wx) were independently germinated on Murashige and Skoog (MS) medium for one week in an incubator at 28 °C/22 °C (day/night). To simulate cold stress, we exposed 7-day-old seedlings to 4 ± 1°C for 4 days in a fridge and allowed them to recover for 4 days. For RNA extraction, we collected the leaves of Dongjin and *sgr* at two time points: 7-day-old seedlings before cold treatment and after 4 days of cold treatment. For confirming diurnal rhythm, we germinated and incubated seeds of japonica rice, cv. Dongjin, on MS medium for a week and transplant the seedlings to a growth chamber (28 °C/25 °C day/night, 14/10 hrs light/dark, and 80% humidity). We then sampled the leaves every two hours in the growth chamber for a day.

### 4.7. RNA Extraction and Quantitative RT-PCR (qRT-PCR) Analysis

Leaf samples from Dongjin and *sgr* before and after cold treatment (four days) were immediately frozen in liquid nitrogen. After total RNA was isolated using the RNAiso (Takara Bio, Shiga, Japan), first-strand cDNA was synthesized using the SuPrimeScript RT Premix (with oligo (dT), 2×) (GeNet Bio, Daegu, Korea). The synthesized cDNAs were amplified using SYBR Green I (GeNet Bio, Korea), and qRT-PCR was performed on the Rotor Gene Q instrument system (Qiagen, Hidden, Germany). To normalize the amplified transcripts, we used a primer pair for *rice ubiquitin 5* (*OsUbi5/Os01g22490*) [122,123]. All the primers for this analysis are summarized in Table S6.

## Figures and Tables

**Table 1:** List of functionally characterized cold-responsive diurnal rhythmic genes (CD genes) in rice.

Locus_ID	Gene_Symbol	Character_Minor	Method ^1^	Detailed Functions	DOI ^2^
**Upregulated cold-responsive diurnal rhythmic genes (Upregulated CD genes)**					
LOC_Os07g22710	*CPK18*	Blast resistance	Knockdown	Binds and phosphorylates MPK5, regulating resistant to blast (*Magnaporthe oryzae*)	[28]
LOC_Os06g45140	*OsbZIP52/RISBZ5*	Cold tolerance	Overexpression	Cold and drought tolerance	[24]
LOC_Os04g58810	*OsCAF1B*	Cold tolerance	Others	Drought tolerance	[25]
LOC_Os09g35030	*OsDREB1A*	Cold tolerance	Overexpression	Cold, drought and salinity tolerance.	[27]
LOC_Os03g17700	*OsMAPK5*	Cold tolerance	Knockdown Overexpression	Resistance to Magnaporthe grisea and Burkholderia glumae. Cold, drought and salinity tolerance.	[26]
LOC_Os03g60080	*SNAC1*	Drought tolerance	Overexpression	Drought and salinity tolerance. Stomatal control.	[29]
LOC_Os07g39220	*OsBZR1*	Dwarfism	Knockdown	Dwarfism. Leaf angle. Brassinosteroid sensitivity.	[30]
LOC_Os03g40540	*OsDWARF*	Dwarf	Mutant	Dwarfism. Brassinosteroid biosynthesis.	[31]
LOC_Os03g08330	*TIFY11b*	Dwarf	Overexpression	Grain size. Plant height	[32]
LOC_Os04g08740	*Etr2*	Flowering	Mutant	Flowering time. Ethylene sensitivity. Stem starch content.	[33]
LOC_Os01g15900	*Rdd1*	Flowering	Knockdown Overexpression	Grain length and width. 1000-grain weight. Flowering time.	[34]
LOC_Os10g42690	*Jmj6*	Panicle flower	Mutant	Number and morphology of floral organ.	[35]
LOC_Os05g41760	*MSF1*	Panicle flower	Mutant	Spikelet determinacy. Floral organ development.	[36]
LOC_Os09g36200	*SGR*	Source activity	Mutant	Leaf senescence. Chlorophyll degradation.	[37]
**Downregulated cold-responsive diurnal rhythmic genes (Downregulated CD genes)**					
LOC_Os05g51830	*HDT701*	Bacterial blight resistance	Knockdown Overexpression	Resistance to *Magnaporthe oryzae* and *Xanthomonas oryzae pv oryzae*.	[38]
LOC_Os09g29820	*OsTFX1*	Bacterial blight resistance	Overexpression	Resistance to *Xanthomonas oryzae pv. oryzae.*	[39]
LOC_Os08g06280	*OsLSD1*	Blast resistance	Knockdown Overexpression	Lesion mimic. Resistance to *Magnaporthe grisea*.	[40]
LOC_Os08g17680	*SDF2-1*	Blast resistance	Knockdown	XA21-mediated resistance to *Xanthomonas oryzae pv. oryzae*	[41]
LOC_Os01g55450	*OsCIPK12*	Drought tolerance	Overexpression	Drought tolerance.	[42]
LOC_Os02g12790	*Cga1*	Dwarf	Knockdown Overexpression	Dwarfism. Tillering. Chlorophyll content. Grain filling rate.	[43]
LOC_Os06g38470	*HDA702*	Dwarf	Knockdown	Elongated uppermost internode. Fertility.	[44]
LOC_Os07g49400	*OsApx2*	Dwarf	Knockdown	Aluminum tolerance. Dwarfism.	[45]
LOC_Os06g04200	*Wx*	Eating quality	Natural variation	Seed amylose content.	[46]
LOC_Os08g07740	*DTH8*	Flowering	Natural variation	Flowering time under long day condition.	[48]
LOC_Os01g08300	*OsCDT3*	Other soil stress tolerance	Knockdown	Aluminum tolerance.	[47]
LOC_Os10g35370	*Fgl*	Source activity	Mutant	Chlorophyll synthesis under high light conditions.	[49]
LOC_Os06g02580	*Zn*	Source activity	Mutant	Chloroplast biosynthesis.	[50]

^1^ Methods used to characterize the function of the genes. ^2^ Digital Object Identifier.

**Table 2:** List of functionally characterized genes in the hypothetical PPI network.

Locus_ID	Gene_Symbol	Character_Minor	Methods ^1^	Detailed Functions	DOI ^2^
LOC_Os04g37920	*Cry1b*	Shoot seedling	Mutant	Leaf sheath elongation during seedling stage. Gibberellin metabolism.	[53]
LOC_Os06g48060	*Star1*	Other soil stress tolerance	Mutant	Aluminum tolerance.	[54]
LOC_Os07g40510	*Bui1*	Dwarf	Mutant	Cell division and expansion. Actin organization.	[55]
LOC_Os11g14220	*Tid1*	Culm leaf	Mutant	Cell division and expansion. Twisted growth. Microtubule arrangement.	[56]
LOC_Os06g07210	*V3*	Source activity	Mutant	Chloroplast development during seedling stage.	[57]
LOC_Os12g08730	*Ostrxm*	Source activity	Knockdown	Chloroplast development. Growth retardation	[58]
LOC_Os07g47300	*ObgC*	Source activity	Mutant	Chloroplast development. Plastid ribosome biogenesis	[59]
LOC_Os11g48070	*Wdl1*	Drought tolerance	Mutant	Cuticle formation. Drought tolerance.	[60]
LOC_Os09g27060	*OsDDM1a*	Dwarf	Knockdown	Dwarfism, DNA methylation	[61]
LOC_Os05g26890	*D1*	Dwarf	Mutant	Dwarfism.	[62]
LOC_Os07g44330	*OsPDK1*	Dwarf	Knockdown	Dwarfism.	[63]
LOC_Os07g06980	*HDA704*	Culm leaf	Knockdown	Dwarfism. Twisted flag leaf.	[44]
LOC_Os07g05620	*OsCIPK23*	Salinity tolerance	Knockdown Overexpression	Fertility. Salinity tolerance.	[64]
LOC_Os06g06750	*OsMADS5*	Panicle flower	Knockdown	Floral organ formation.	[65]
LOC_Os09g03610	*rFCA*	Flowering	Overexpression	Flowering time.	[66]
LOC_Os07g49460	*OsPRR37*	Flowering	Natural variation	Flowering time.	[67]
LOC_Os03g51030	*OsPhyA*	Flowering	Mutant	Flowering time. Deetiolation response. Sensitivity to red and far-red light.	[68]
LOC_Os03g19590	*OsPhyB*	Flowering	Mutant	Flowering time. Deetiolation response. Sensitivity to red and far-red light.	[68]
LOC_Os03g54084	*OsPhyC*	Flowering	Mutant	Flowering time. Deetiolation response. Sensitivity to red and far-red light.	[68]
LOC_Os08g04270	*OsFIE2*	Germination dormancy	Knockdown	Grain size. Grain filling rate. Seed dormancy.	[69]
LOC_Os04g25540	*S28*	Sterility	Natural variation	Hybrid sterility between *Oryza sativa* and *Oryza glaberrima*. Pollen development. Interaction with S27	[70]
LOC_Os02g53140	*Pps*	Dwarf	Mutant	Juvenile to adult phase change. Flowering time independent of daylength. Dwarfism.	[71]
LOC_Os07g32480	*BRK1*	Sterility	Mutant	Meiosis.	[72]
LOC_Os07g28280	*Rurm1*	Others	Mutant	Mobilization of the Active MITE mPing	[73]
LOC_Os12g10540	*OsMADS13*	Panicle flower	Mutant	Ovule identity.	[74]
LOC_Os01g72090	*Se13*	Flowering	Mutant	Photoperiodic response.	[75]
LOC_Os09g38030	*Ugp1*	Dwarf	Knockdown	Pollen callose deposition. Dwarfism.	[76]
LOC_Os05g28510	*OsImp* *β1*	Sterility	Mutant	Pollen tube elongation.	[77]
LOC_Os06g04280	*Epsps*	Panicle flower	Others	production of panicle	[78]
LOC_Os12g43550	*Gpa1*	Eating quality	Mutant	Pro-gultelin content in seed. Floury endosperm.	[79]
LOC_Os06g06090	*OsMAPK6*	Others	Knockdown	Regulation of stress response genes.	[80]
LOC_Os03g57450	*OsCPK10*	Blast resistance	Overexpression	Resistance to *Magnaporthe grisea*.	[81]
LOC_Os04g38480	*OsSERK1*	Blast resistance	Overexpression	Resistance to *Magnaporthe grisea*.	[82]
LOC_Os09g25070	*OsWRKY62*	Bacterial blight resistance	Overexpression	Resistance to *Xanthomonas oryzae pv. oryzae*.	[83]
LOC_Os07g25710	*OsPHR2*	Other soil stress tolerance	Overexpression	Response to phosphate starvation.	[84]
LOC_Os07g10830	*OsDGL1*	Root	Mutant	Root development.	[85]
LOC_Os05g49890	*OsRan2*	Other stress resistance	Knockdown Overexpression	Salinity and osmotic stress tolerance. ABA sensitivity.	[86]
LOC_Os02g33770	*OsGTgamma-1*	Salinity tolerance	Mutant	Salinity tolerance.	[87]
LOC_Os05g45420	*OsSnRK1a*	Germination dormancy	Mutant	Seed germination. Seedling growth.	[88]
LOC_Os11g09280	*PDIL1*	Eating quality	Mutant	Starch biosynthesis.	[89]
LOC_Os03g51600	*Rip-3 (a-tubulin)*	Panicle flower	Others	suppress panicle elongation during water deficit	[90]
LOC_Os07g34570	*OsDR8*	Bacterial blight resistance	Knockdown	Thiamine mediated resistance to *Xanthomonas oryzae* and *Magnaporthe grisea*.	[91]

^1^ Methods used to characterize the function of the genes. ^2^ Digital Object Identifier.

## Supplementary Materials

Supplementary materials can be found at https://www.mdpi.com/1422-0067/21/18/6872/s1.
