# Supplementary material for: Systematic Analysis of Cold Stress Response and Diurnal Rhythm Using Transcriptome Data in Rice Reveals the Molecular Networks Related to Various Biological Processes
Source: Int J Mol Sci. 2020 Sep 19;21(18):6872. doi: 10.3390/ijms21186872 (PMC7554834; doi:10.3390/ijms21186872)
Supplement: Supplementary file 1 [file ijms-21-06872-s001.zip › supporting information.docx]

**Supporting Information**

**Figure S1. MapMan analysis of the CD genes.**

(A) Results of mapping 465 genes to Metabolism overview. (B) Regulation overview. Red squares, up-regulated genes; green squares, down-regulated genes. Additionally, box areas with red lines in metabolism and regulation overview highlight that are discussed in the comprehensive results.

**Figure S2. Raw PPI network retrieved from the Rice Interaction Viewer.**

The raw PPI network compromising 764 nodes and 1,340 edges by querying 465 CD genes.

**Figure S3. Phenotypic comparison between Hwacheong-wx rice and *sgr* mutant under normal and cold stress conditions.**

Phenotypic comparison between Hwacheong-wx rice and sgr mutant under cold stress treatment. White bar indicates 2 cm.

**Table S1. List of cold-regulated genes and their expression values.**

**Table S2. List of cold-regulated and circadian rhythmic genes.**

**Table S3. Summary of enriched GO terms in the cold-regulated and circadian rhythmic genes.**

**Table S4. Summary of functional classification of the cold-induced rhythmic genes.**

**Table S5. Summary of network information.**

**Table S6. Summary of primers used in this study.**
