# Supplementary figures and images for: Systematic Analysis of Cold Stress Response and Diurnal Rhythm Using Transcriptome Data in Rice Reveals the Molecular Networks Related to Various Biological Processes
Source: Int J Mol Sci. 2020 Sep 19;21(18):6872. doi: 10.3390/ijms21186872 (PMC7554834; doi:10.3390/ijms21186872)

A

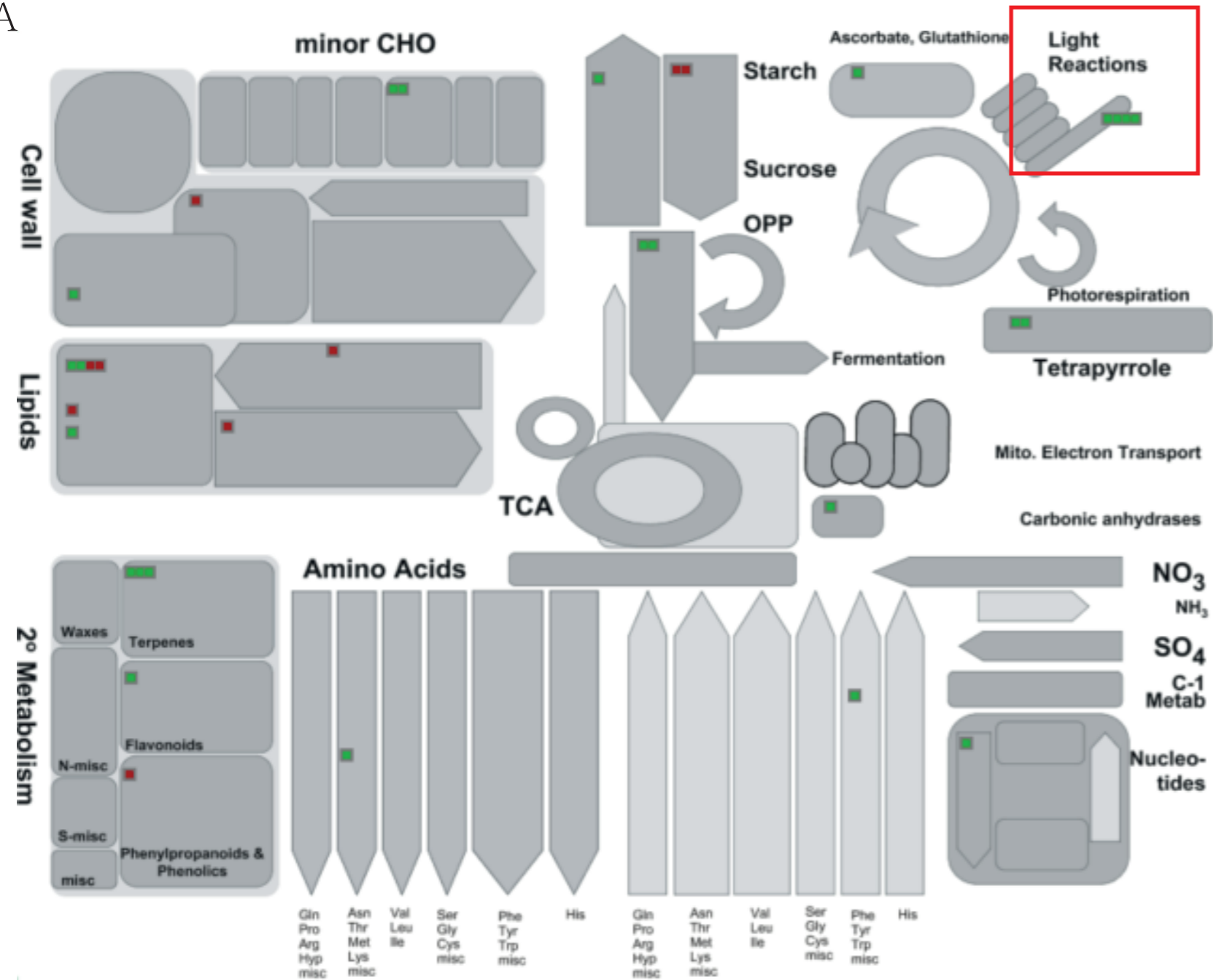

B

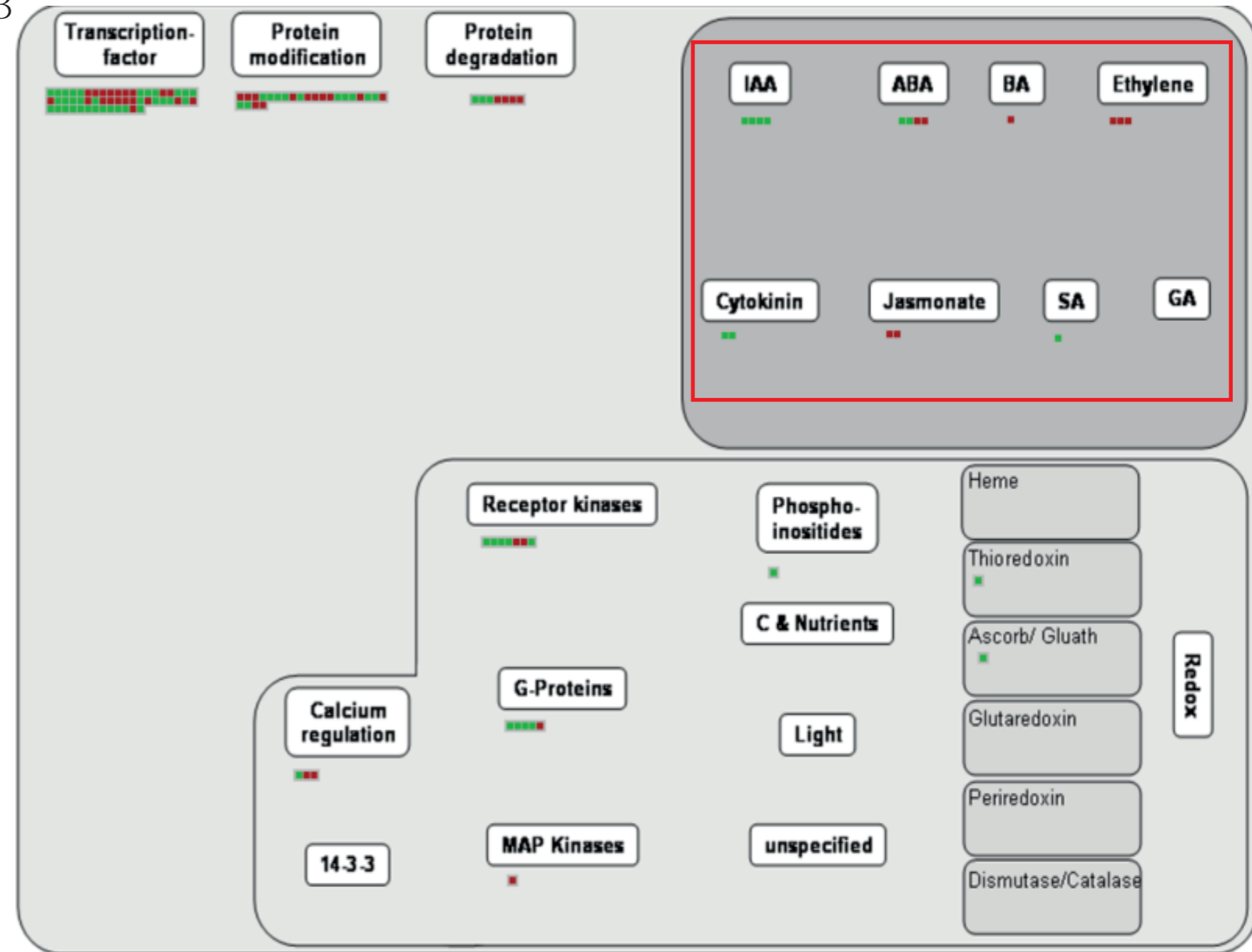

Supplement: Supplementary file 1 [file ijms-21-06872-s001.zip › Figure S1_2nd_revision.pdf]

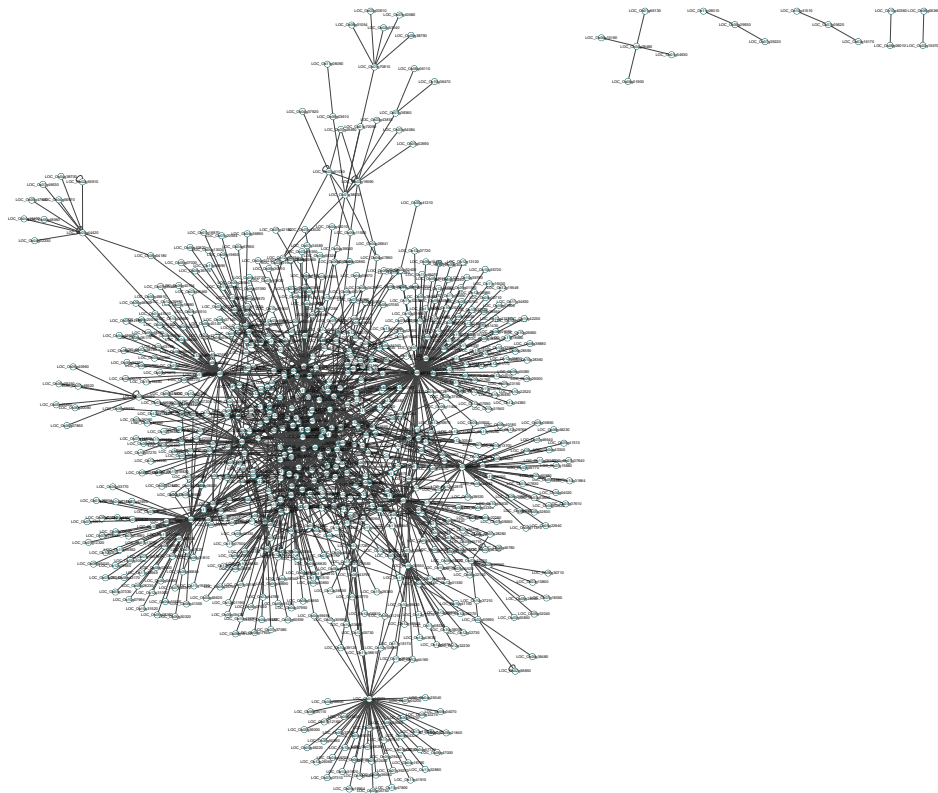

Supplement: Supplementary file 1 [file ijms-21-06872-s001.zip › Figure S2_2nd_revision.pdf]

Cold 0 day

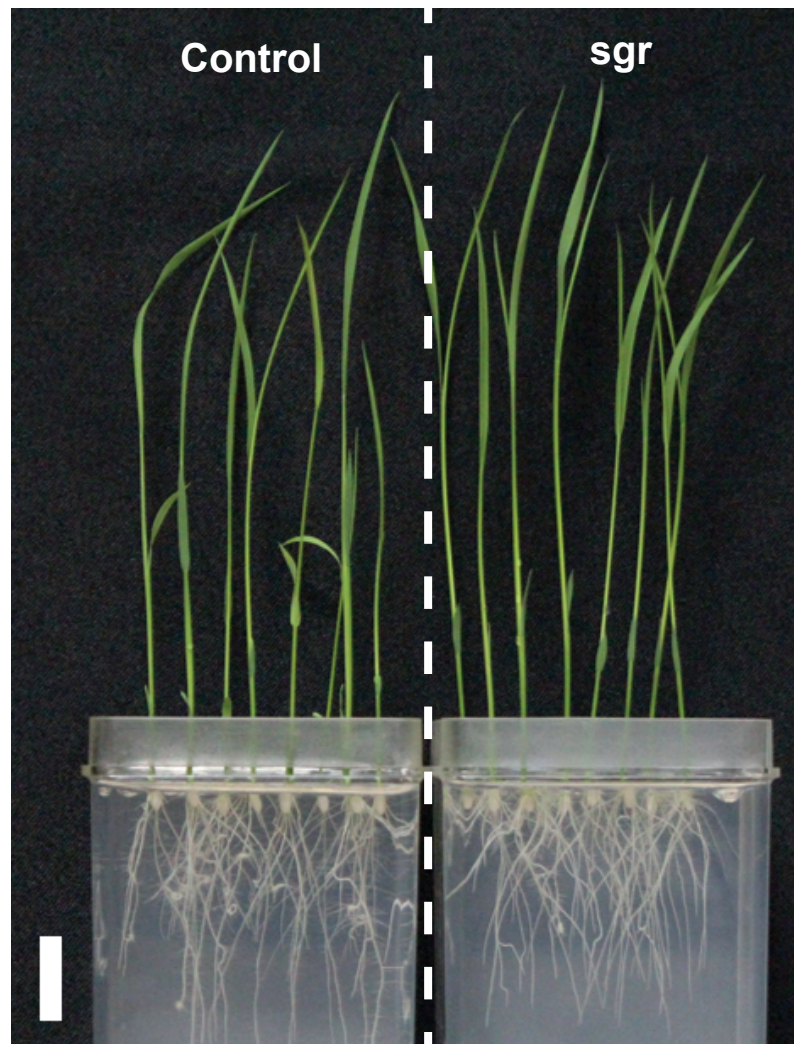

Cold 4 days

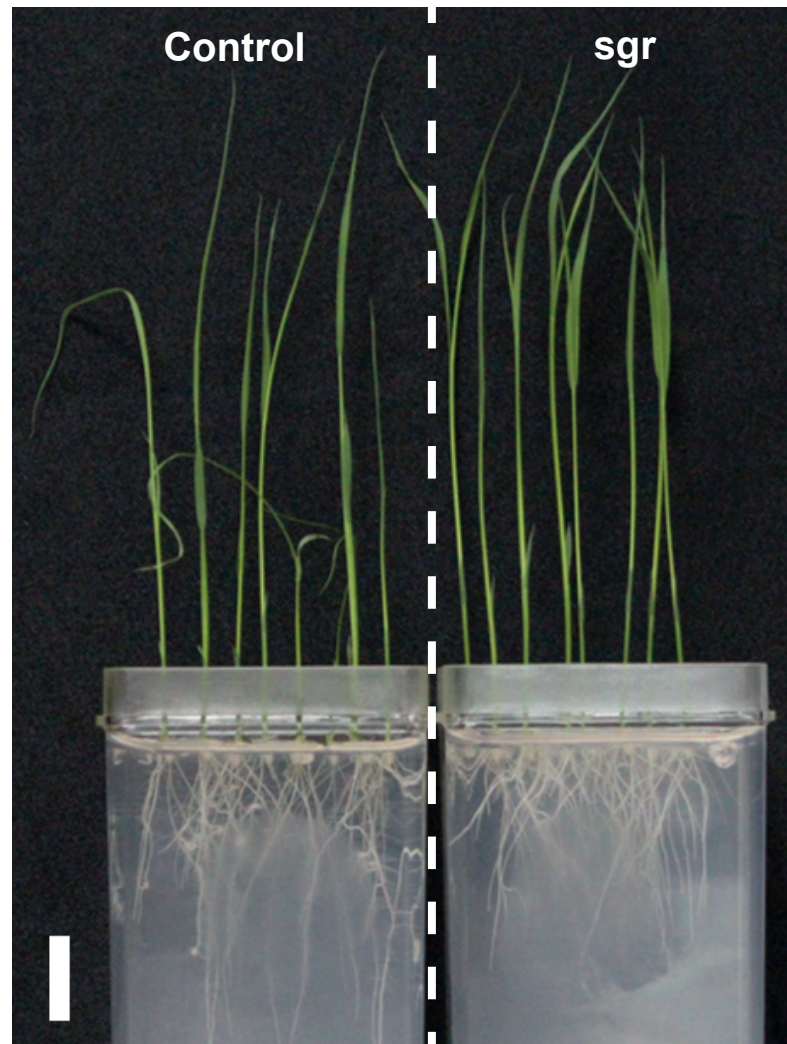

Recovery 5 days

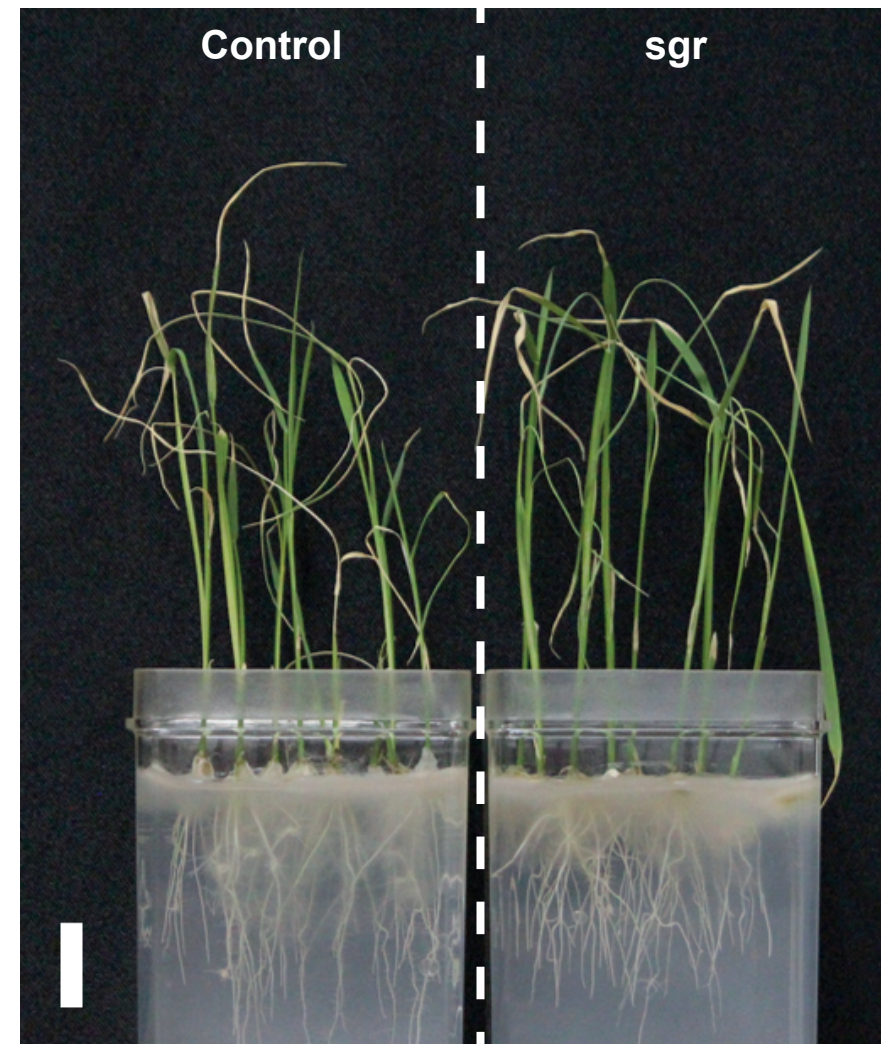

Supplement: Supplementary file 1 [file ijms-21-06872-s001.zip › Figure S3_2nd_Rev_2.pdf]
